# Meibomian Glands and Tear Film Findings in Type 2 Diabetic Patients: A Cross-Sectional Study

**DOI:** 10.3389/fmed.2022.762493

**Published:** 2022-04-05

**Authors:** Huping Wu, Xie Fang, Shunrong Luo, Xumin Shang, Zhiwen Xie, Nuo Dong, Xianwen Xiao, Zhirong Lin, Zuguo Liu

**Affiliations:** ^1^Eye Institute and Affiliated Xiamen Eye Center of Xiamen University, School of Medicine, Xiamen University, Xiamen, China; ^2^Fujian Provincial Key Laboratory of Ophthalmology and Visual Science, Xiamen, China; ^3^Fujian Key Laboratory of Ocular Surface and Corneal Diseases, Affiliated Xiamen Eye Center of Xiamen University, Xiamen, China

**Keywords:** diabetes, glycosylated hemoglobin, meibomian gland dysfunction, dry eye, ocular discomfort

## Abstract

**Background:**

The characteristics of the meibomian gland and tear film in patients with type 2 diabetes (T2D) with different glycemic control levels and diabetic durations remain largely unexplored. This study aimed to identify the association of dry eye and meibomian gland dysfunction (MGD) in T2D.

**Materials and Methods:**

Ninety-nine patients with type 2 diabetes mellitus (DM group), 33 dry eye patients without diabetes mellitus (DE group), and 40 normal subjects (NC group) were recruited for this study. Participants were evaluated with an Ocular Surface Disease Index (OSDI) questionnaire, tear film breakup time (BUT), the Schirmer I test (SIT), corneal fluorescein staining (FL), lipid layer thickness (LLT), and MGD parameters. Glycosylated hemoglobin (HbA_1*c*_) and duration of diabetes were recorded.

**Results:**

The SIT value in the DM group was higher than that of the DE group (*p* < 0.05). The BUT and LLT were lower, and MGD parameters were higher in the DM group than those of the DE and NC groups (*p* < 0.05). In the DM group, 47 patients were diagnosed with dry eye (DM + DE group), whereas 40 patients without dry eye were categorized as the DM − DE group. The SIT, BUT, and LLT values in the DM − DE group were higher (*p* < 0.01), and MGD parameters were lower (*p* < 0.01) in the DM − DE group than those of the DM + DE group. The MGD parameters were higher in the DM − DE group than those in the NC group (*p* < 0.05). The HbA_1*c*_ levels were correlated with OSDI, BUT, LLT, FL, and MGD parameters (*p* < 0.001) in the DM group. However, in patients with low HbA_1*c*_, normal SIT value, and low OSDI, the MGD parameters were higher than those in the NC group (*p* < 0.05). The duration of diabetes positively correlated with MGD parameters (*p* < 0.001).

**Conclusion:**

Asymptomatic MGD may be an early sign of dry eye and ocular discomfort in T2D. The MGD parameters were associated with the HbA_1*c*_ level and diabetic duration.

## Introduction

Ocular function disruption is one of the most common complications in diabetes (1). Diabetic cataract, diabetic retinopathy, neovascular glaucoma, etc., are the common complications leading to blindness (2). Furthermore, dry eye is very common in patients with diabetes, presenting with a foreign body sensation, dryness, burning sensation, etc., that affects their daily lives and their ability to work (3). The incidence of dry eye has been reported to range between 27.7 and 54.3% in patients with type 2 diabetes (T2D) (4–7). Lacrimal functional unit dysfunction, abnormal tear dynamics, diabetic neuropathy, and tear film dysfunction are considered to be the major etiologies of diabetes mellitus-associated dry eye (5). Meibomian gland dysfunction (MGD) results in abnormal tear film layer and ocular discomfort (8), which is considered to be the main cause of evaporative dry eye (8). Dry eye in T2D has been extensively reported. However, the association of dry eye and MGD in T2D is still unclear. Some study found that the glycosylated hemoglobin (HbA_1*c*_) levels correlated with the presence of dry eye in patients with diabetes (4, 9).

Hence, we hypothesized that MGD was one of the missing links between diabetes and dry eye, and MGD might be critical in the pathogenesis of dry eye in T2D. This study aims to investigate the morphological and functional characteristics of meibomian gland and their roles in T2D with or without dry eye.

## Materials and Methods

### Subjects and Criteria

This study was approved by the Ethics Committee of Xiamen Eye Center, an affiliate of Xiamen University. All methods below were performed in accordance with the relevant guidelines and regulations. Written informed consent was obtained from participants in this study in accordance with the Declaration of Helsinki and its subsequent revision. Informed consent for online open-access publication of images or information from participants was also obtained.

Ninety-nine patients who were diagnosed with type 2 diabetes [according to the 1999 WHO diagnostic criteria (10) for diabetes] were recruited as the diabetes mellitus group (DM group). Thirty-three patients without diabetes who were diagnosed with dry eye (11–13) [Ocular Surface Disease Index (OSDI)] ≥ 13 and tear film breakup time (BUT) < 5 s were recruited as the dry eye group (DE group). Meanwhile, 40 normal individuals were recruited as normal control (NC group). The subjects of the NC group have no history of ocular disease or systemic pathology that could affect the test results, have no history of topical medication within the last 6 months, and have no signs or symptoms of ocular surface disease. All the examinations were performed during the period from 1 October, 2016 to 31 December, 2016 in the Affiliated Xiamen Eye Center of Xiamen University, Xiamen, China. Patients with blepharitis, allergic conjunctivitis, infectious keratoconjunctivitis, pterygium, eye trauma, eye operation, eye continuous medication history, or corneal contact lens wearing history were excluded from this study. Meanwhile, patients with systemic diseases, such as Sjogren’s syndrome, Stevens–Johnson syndrome, hyperthyroidism, and pemphigoid, were excluded.

### Ocular Surface Disease Index Questionnaire

The degree of ocular discomfort was evaluated by an OSDI questionnaire in all subjects. OSDI (14) evaluates 12 symptoms, and every score was calculated according to the duration of the symptoms with 100 points in total. The scale is graded as follows: normal: 10–12 points, mild: 13–22 points, moderate: 23–33 points, and severe: 33–100 points. Subjects with dry eye have at least mild ocular surface symptomology, which means OSDI ≥ 13 points (15).

### Clinical Examinations

A series of dry eye parameters were performed in the following order: lipid layer thickness (LLT), tear breakup time (BUT), corneal fluorescein staining scoring (FL), and Schirmer I test (SIT). MGD parameters were evaluated. All the tests were carried out by the same masked investigator. Only the values of one eye (right or left eye, randomly selected by simple randomization using a table of random digit) were included in this study. The HbA_1*c*_ level was also tested in all subjects.

### Assessment of Tear Film Parameters

The tear LLT test was performed using an interferometer (Lipiview™, TearScience Inc., Morrisville, NC, United States). The patient was asked to blink naturally when tested, and tear film images were captured for 30–60 s (16).

For the BUT test, the sodium fluorescein filter paper was applied to the center of the lower eyelid causing fluorescein sodium to flow into the conjunctival sac. After blinking for three times, the test was conducted by inspecting with a slit lamp under low brightness of a cobalt blue light (BQ900IM9900, Haag-Streit, Switzerland). The average tear BUT of three repeated measurement was considered the BUT of one eye (seconds). Corneal FL was scored under cobalt blue light by the fluorescent paper after a corneal surface of patient was stained for 90 s as mentioned in the BUT test. Cornea was graded as follows: 0: no staining, 1: slight scatted staining, 2: moderate staining between 1 and 3, and 3: severe staining (17).

For SIT, the tear filter paper strip was placed in the conjunctival sac about 1/3 beneath the eyelid center, and the length of filter strip wetting (mm) was read after 5 min.

### Evaluation of Meibomian Gland Parameters

Lid margin abnormalities (lid margin score) were assessed with a slit lamp as follows (18): irregular lid margins, score 1; vascular engorgement, score 2; glandular orifice obstruction, score 3; and anterior or posterior displacement of the mucocutaneous junction, score 4. No presence of above 4 signs was indicated as score 0.

The meibomian gland yielding liquid secretion score (MGYLS score) was graded under the diffusing light of a slit lamp. A constant pressure about 20 kPa was applied to the lower eyelid using a meibomian gland evaluator (MGE, TearScience Inc., Morrisville, NC, United States) for 10 s, and then the orifice opening of five consequent glands where pressure was applied were evaluated. The number of obstructed openings of the total 15 glands in three different locations (nasal, central, and temporal sides) was inspected and recorded. The MGYLS score of each eye was the average number of obstructed glands of the upper and lower eyelids (15 points in total). The level of MGYLS was graded as follows: grade 0, 0–3 points; grade 1, 4–7 points; grade 2, 8–11 points; and grade 3, 12–15 points.

The qualitative appearance of lipid secretion of each meibomian gland (meibum score) was graded as follows: 0 point (clear or slight yellow liquid secretion); 1 point (creamy yellow or cloudy liquid secretion); 2 points (granular in liquid with white and/or yellow color); and 3 points (toothpaste shape) (19). The total 15 glands in three different locations (nasal, central, and temporal sides) were inspected and recorded. The total score of each eye was 0–90, including the upper and lower eyelids. The level of the meibum score was graded as follows: grade 0, 0–3 point; grade 1, 4–10 points; grade 2, 11–30 points; grade 3, 31–60 points; and grade 4, 61–90 points.

The loss of meibomian glands (meiboscore) was examined using a non-contact infrared meibography (20, 21) (Keratograph 5M, OCULUS, Wetzlar, Germany). Meiboscore was graded as follows: 0 point (no absence); 1 point (absence of less than 1/3 of total glands); 2 points (absence of more than 1/3 but less than 2/3 of total glands); and 3 points (absence of more than 2/3 of total glands). Upper and lower eyelids are 0–6 points in sum. Examinations were performed by the same masked ophthalmologist.

The subject was diagnosed as MGD according to the criteria briefly as follows (18, 22): the presence of lid margin abnormality (lid margin score ≥ 1), and/or an altered quality of expressed secretions (meibum grade ≥ 1), and/or a decreased or absent expression (MGYLS grade ≥ 1). The subject with MGD could be further diagnosed as “asymptomatic MGD” if he/she had no symptoms (OSDI < 13) (23, 24).

### Statistical Analysis

All data were analyzed using software SPSS 26.0 (SPSS Inc., Chicago, IL, United States). Measurement data were presented as mean and standard deviation. Difference of sex ratio was evaluated by the chi-square test. Differences in other parameters among groups were analyzed by using the one-way ANOVA test. The Dunn–Bonferroni test was used to make a pairwise comparison between two groups. For non-parametric data (corneal FL score and meibomian gland parameters), a Kruskal–Wallis ANOVA test along with the *post hoc* test was used for multiple comparisons. The correlation between the HbA_1*c*_ level and various factors was estimated by the Spearman rank correlation analysis. For the secondary analysis, patients with diabetes (DM group) were further subgrouped according to the dry eye condition, the HbA_1*c*_ level, and the duration of diabetes. A multivariate analysis was performed to identify the variables that most correlated with any of the meibomian gland parameters in the DM group. The differences were regarded statistically significant when the value of *p* was <0.05.

## Results

Demographic characteristics of subjects are presented in Table 1.

**TABLE 1:** Demography of the subjects.

	NC group (*n* = 40)	DE group (*n* = 33)	DM group (*n* = 99)	*p* value
Age (years)	58.55 ± 7.18	59.09 ± 7.25	59.72 ± 6.05	0.434
Sex ratio (M/F)	21/19	15/18	47/52	> 0.05
Blood glucose (mmol/L)	5.77 ± 0.39	5.72 ± 0.44	7.99 ± 1.64	< 0.001
HbA_1*c*_ (%)	5.91 ± 0.19	5.96 ± 0.28	7.37 ± 1.28	< 0.001
Duration (years)	/	/	5.24 ± 3.06	/

*NC, normal control; DE, dry eye; DM, diabetes mellitus; n, number of participants; M/F, male/female. All data are presented as mean ± standard deviation.*

### Dry Eye and Ocular Discomfort in Patients With Type 2 Diabetes

The tear film parameters and the meibomian gland parameters of subjects are shown in Table 2. The OSDI score of the NC group was significantly lower than those of both the DE group and the DM group (*p* < 0.001). Meanwhile, the OSDI score of the DM group was also significantly lower than that of the DE group (*p* = 0.016). Furthermore, the average SIT, BUT, and tear LLT values in both DE and DM groups were all significantly lower (*p* < 0.001) than those in the NC group, whereas the values of meibomian gland parameters (except lid margin score) were all higher (*p* < 0.001) than those in the NC group (Table 2).

**TABLE 2:** The tear film and meibomian gland parameters in different groups.

	NC group (*n* = 40)	DE group (*n* = 33)	DM group (*n* = 99)	*p* value
OSDI	10.31 ± 1.45	24.94 ± 5.22*^a^*	19.98 ± 8.91^a^	<0.001
Schirmer I (mm)	13.58 ± 2.92	5.14 ± 2.12^a^	9.59 ± 3.17^ab^	<0.001
BUT (s)	10.87 ± 1.79	4.80 ± 0.83^a^	6.25 ± 1.99^a^	<0.001
FL score	0.28 ± 0.45	0.91 ± 0.52^a^	0.67 ± 0.64	<0.05
LLT (nm)	79.18 ± 8.00	63.86 ± 8.04^a^	59.55 ± 9.34^ab^	<0.001
Lid margin score	1.38 ± 0.63	1.57 ± 0.49	2.03 ± 0.76^ab^	<0.001
MGYLS score	3.33 ± 1.07	4.31 ± 0.80^a^	6.28 ± 1.88^ab^	<0.001
Meibum score	14.53 ± 4.14	19.85 ± 5.27^a^	25.01 ± 6.14^ab^	<0.001
Meiboscore	2.25 ± 0.81	3.16 ± 1.05^a^	3.53 ± 1.01^ab^	<0.001

*NC, normal control; DE, dry eye; DM, diabetes mellitus; n, number of participants; OSDI, ocular surface disease index; BUT, tear breakup time; FL, corneal fluorescein staining; LLT, lipid layer thickness; MGYLS, meibomian gland yielding liquid secretion. All data are presented as mean ± standard deviation. ^a^p < 0.05 compared to the NC group. ^b^p < 0.05 compared to the DE group.*

The average SIT value in the DM group (9.59 ± 3.17 mm) was significantly higher than that in the DE group (5.14 ± 2.12 mm).

### Meibomian Gland Dysfunction in Patients With Type 2 Diabetes

The four MGD parameters in the DM group were all significantly higher (*p* < 0.001) than those of the other two groups (Table 2). Although the average SIT value of the DM group is higher than that of the DE group, the severity of MGD is more apparent in the DM group. Interestingly, all the subjects in DE and DM groups could be eventually diagnosed as MGD according to the diagnostic criteria.

**FIGURE 1:**
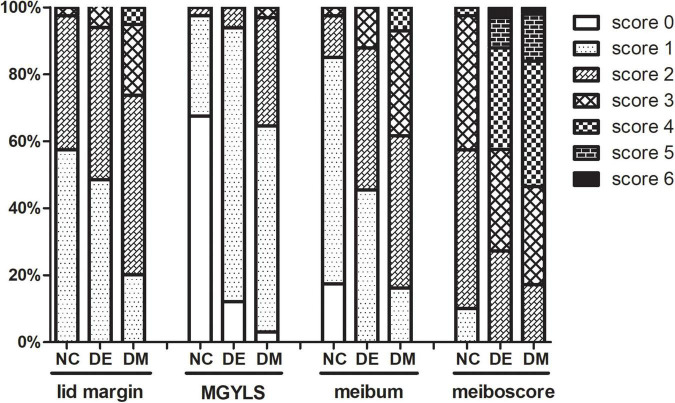
The stacked percentage column chart showing the detailed distribution of meibomian gland parameters. NC, normal control; DE, dry eye; DM, diabetes mellitus; MGYLS, meibomian gland yielding liquid secretion.

The detailed distribution of meibomian gland parameters in different groups is shown in Figure 1. Twenty-six patients (26.3%) in the DM group had a high lid margin score (score ≥ 3), whereas two patients (6.1%) in the DE group and only one patient (2.5%) in the NC group had high score. A high level of the MGYLS grade (grade ≥ 2) was observed in 35 patients (35.4%) in the DM group, whereas only two patients (6.1%) in the DE group and one patient (2.5%) in the NC group were observed. High meibum grade (grade ≥ 3) was seen in 38 patients (38.4%) in the DM group, whereas only four patients (12.1%) in the DE group and only one patient (2.5%) in the NC group were seen. A high meiboscore (score ≥ 3) was seen in 82 patients (82.8%) in the DM group and 24 patients (72.7%) in the DE group, whereas only 17 patients (42.5%) in the NC group were seen.

### Presence of Meibomian Gland Dysfunction in Patients With Diabetes Without Dry Eye

According to the recently published article (11), the existence of ocular symptoms and BUT for less than 5 s was proposed as the diagnostic criteria for dry eye. Based on the values of OSDI (≥13) and BUT (<5 s), 47 patients with diabetes (47.5%) in the DM group that were diagnosed with dry eye were marked as the DM + DE group, whereas 40 patients (40.4%) without dry eye served as the DM − DE group (OSDI < 13 and BUT > 5 s). Interestingly, the SIT and LLT in the DM − DE group were significantly higher (*p* < 0.01) than those of the DM + DE group. The values of four meibomian gland parameters, the OSDI score, and the FL score were significantly lower (*p* < 0.01) in the DM − DE group than those of the DM + DE group, although no significant difference of BUT was found. More importantly, the values of four meibomian gland parameters were significantly higher in the DM − DE group than those of the NC group, although the OSDI were below 13 in both groups. In fact, the values of DM − DE group met the diagnostic criteria of asymptomatic MGD. This suggested that asymptomatic MGD might emerge before the ocular discomfort develops in T2D (Table 3).

**TABLE 3:** Tear film and meibomian gland parameters in patients with T2DM with or without dry eye.

	NC group (*n* = 40)	DE group (*n* = 33)	DM − DE group (*n* = 40)	DM + DE group (*n* = 47)	*p*-value
Age (yr)	58.55 ± 7.18	59.09 ± 7.25	59.45 ± 4.91	61.5 ± 6.56	>0.05
Sex ratio (M/F)	21/19	15/18	17/23	19/23	>0.05
HbA_1*c*_ (%)	5.91 ± 0.19	5.96 ± 0.28	6.65 ± 0.65^ac^	8.16 ± 1.37^abc^	<0.001
OSDI	10.31 ± 1.45	24.94 ± 5.22	10.96 ± 1.44^c^	27.90 ± 6.06^ab^	<0.001
Schirmer I (mm)	13.58 ± 2.92	5.14 ± 2.12	11.25 ± 2.61^ac^	7.67 ± 2.84^abc^	<0.001
BUT (s)	10.87 ± 1.79	4.80 ± 0.83	6.99 ± 1.83^ac^	4.83 ± 1.43^a^	<0.001
FL score	0.28 ± 0.45	0.91 ± 0.52	0.48 ± 0.55^c^	0.98 ± 0.66^ab^	<0.001
LLT (nm)	79.18 ± 8.00	63.86 ± 8.04	63.76 ± 9.78^a^	54.96 ± 7.69^abc^	<0.001
Lid margin score	1.38 ± 0.63	1.57 ± 0.49	1.71 ± 0.72^a^	2.39 ± 0.69^abc^	<0.001
MGYLS score	3.33 ± 1.07	4.31 ± 0.80	5.53 ± 1.58^ac^	7.21 ± 1.92^abc^	<0.001
Meibum score	14.53 ± 4.14	19.85 ± 5.27	22.31 ± 5.49^a^	28.33 ± 5.63^abc^	<0.001
Meiboscore	2.25 ± 0.81	3.16 ± 1.05	3.11 ± 0.91^a^	4.0 ± 0.96^abc^	<0.001

*NC, normal control; DM − DE, diabetes mellitus without dry eye; DM + DE, diabetes mellitus with dry eye; n, number of participants; M/F, male/female; OSDI, ocular surface disease index; BUT, tear breakup time; FL, corneal fluorescein staining; LLT, lipid layer thickness; MGYLS, meibomian gland yielding liquid secretion. All data are presented as mean ± standard deviation. ^a^p < 0.05 compared to the NC group. ^b^p < 0.05 compared to the DM − DE group. ^c^p < 0.05 compared to the DE group.*

It was also noted that subjects in the DM + DE and DE groups were represented with similar ODSI, BUT, and FL scores. However, the values of SIT and all of the four MGD parameters were significantly higher (*p* < 0.05), whereas the LLT was lower (*p* < 0.05) in the DM + DE group than those of the DE group, indicating the dry eye condition in T2D might mainly originate from the impairment of the meibomian gland function (Table 3). In DM − DE group, the OSDI and FL scores were lower (*p* < 0.05), whereas the SIT and BUT values and the MGYLS score were higher (*p* < 0.05) than those in the DE group. No significant difference in LLT, the lid margin score, the meibum score, and meiboscore were found between the DM − DE and DE groups (*p* > 0.05, Table 3).

In order to gain insight into the importance of the glycemic control level in dry eye in patients with diabetes, the patients were further separated into several subgroups according to HbA_1*c*_ levels. The DM − DE group was further separated into two subgroups: the DM − DE-1 subgroup (HbA_1*c*_ < 7%) and the DM − DE-2 subgroup (HbA_1*c*_ ≥ 7%); the DM + DE group was separated into three subgroups: the DM + DE-1 subgroup (HbA_1*c*_ < 7%), the DM + DE-2 subgroup (7% ≤ HbA_1*c*_ < 9%), and the DM + DE-3 subgroup (HbA_1*c*_ ≥ 9%). Table 4 shows the tear film and meibomian gland parameters of the subgroups and the NC group at different HbA_1*c*_ levels.

**TABLE 4:** Tear film and meibomian gland parameters at different HbA_1*c*_ levels.

		DM − DE group		DM + DE group		
			
	NC group (*n* = 40)	DM − DE-1 HbA_1*c*_ < 7% (*n* = 26)	DM − DE-2 HbA_1*c*_ ≥ 7% (*n* = 14)	DM + DE-1 HbA_1*c*_ < 7% (*n* = 17)	DM + DE-2 7 ≤ HbA_1*c*_ < 9% (*n* = 15)	DM + DE-3 HbA_1*c*_ ≥ 9% (*n* = 15)
Age(yr)	58.55 ± 7.18	58.92 ± 4.71	60.43 ± 5.32	57.7 ± 6.57	61.34 ± 6.38	64.60 ± 5.10
HbA_1*c*_ (%)	5.91 ± 0.19	6.22 ± 0.25	7.45 ± 0.32	6.51 ± 0.34	7.89 ± 0.42	9.76 ± 0.38
OSDI	10.31 ± 1.45	10.60 ± 1.42	11.71 ± 1.22	22.68 ± 3.33	27.52 ± 4.99	32.47 ± 5.22
Schirmer I (mm)	13.58 ± 2.92	11.90 ± 2.49	10.04 ± 2.27	9.33 ± 2.46	8.2 ± 2.77	5.80 ± 2.11
BUT (s)	10.87 ± 1.79	7.65 ± 1.63	5.77 ± 1.56	5.89 ± 1.06	4.93 ± 1.34	3.87 ± 1.13
FL score	0.28 ± 0.45	0.38 ± 0.49	0.64 ± 0.62	0.71 ± 0.62	0.90 ± 0.61	1.27 ± 0.64
LLT (nm)	79.18 ± 8.00	66.40 ± 7.25	58.89 ± 7.41	61.46 ± 6.95	55.6 ± 5.67	49.13 ± 5.75
Lid margin score	1.38 ± 0.63	1.65 ± 0.68	1.82 ± 0.77	1.75 ± 0.67	2.01 ± 0.64	2.60 ± 0.67
MGYLS score	3.33 ± 1.07	5.53 ± 1.57	5.68 ± 1.69	5.87 ± 1.73	7.30 ± 1.61	8.20 ± 1.77
Meibum score	14.53 ± 4.14	21.92 ± 5.03	23.04 ± 6.29	24.29 ± 6.10	28.21 ± 4.16	31.70 ± 4.34
Meiboscore	2.25 ± 0.81	3.02 ± 0.90	3.29 ± 0.94	3.50 ± 0.93	3.97 ± 0.96	4.43 ± 0.77

*NC, normal control; DM − DE, diabetes mellitus without dry eye; DM + DE, diabetes mellitus with dry eye; n, number of participans; OSDI, ocular surface disease index; BUT, tear breakup time; FL, corneal fluorescein staining; LLT, lipid layer thickness; MGYLS, meibomian gland yielding liquid secretion. All data are presented as mean ± standard deviation.*

As the HbA_1*c*_ levels increased in the three DM + DE subgroups, the OSDI and FL scores also increased, whereas the SIT, BUT, and tear LLT values decreased (Table 4). Meanwhile, the scores of meibomian gland parameters gradually increased. In comparison with the NC group (13.58 ± 2.92 mm), the SIT value of the DM + DE group with good glycemic control (DM + DE-1 group) was decreased (9.33 ± 2.46 mm, *p* < 0.01), but was still close to the normal cut-off value (10 mm). In contrast, the SIT value of the DM + DE group with poor glycemic control (DM + DE-3 group) significantly decreased (5.80 ± 2.11 mm, *p* < 0.001). In addition, the tear BUT, FL, LLT scores, and all meibomian gland parameters of the DM + DE group with good glycemic control (DM + DE-1 group) were significantly abnormal when compared to the normal controls (*p* < 0.001).

**FIGURE 2:**
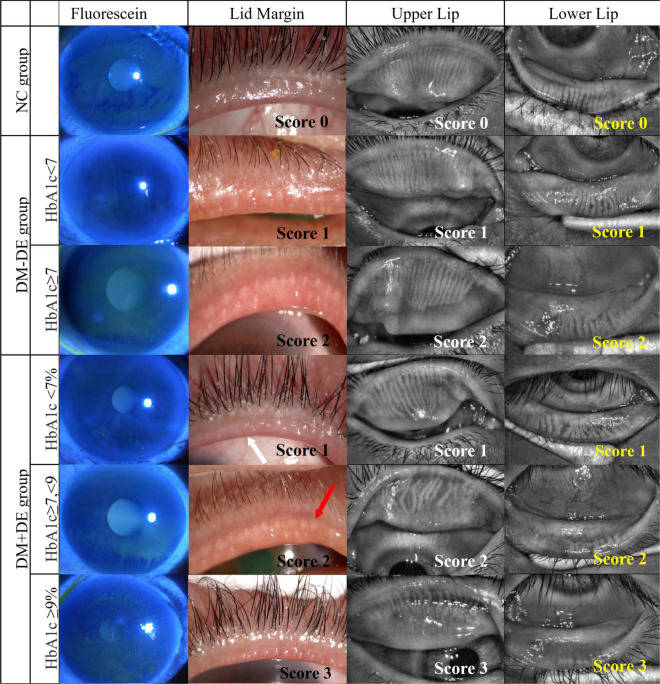
Representative images of corneal fluorescein staining, lid margin, and meibography of upper lid and lower lid in the six subgroups with different HbA_1*c*_ levels. NC, normal control; DM – DE, diabetes mellitus without dry eye; DM + DE, diabetes mellitus with dry eye. The first row of panels shows a normal eye with score 0; The second row shows the right eye of a 52-year-old female from the DM – DE group with good glycemic control, HbA_1*c*_: 6.1%, OSDI:25; BUT: 6.7 s, Schirmer I test (SIT): 13 mm; meibum grade was 1. The third row shows the left eye of a 63-year-old male in the DM – DE group with poor glycemic control: HbA_1*c*_: 7.8%; OSDI: 32; BUT: 5.5 s; SIT: 9.5 mm; meibum grade was 2. The fourth row shows the left eye of a 40-year-old female in the DM + DE group with good glycemic control: HbA_1*c*_: 6.1%; OSDI: 18; BUT: 4.8 s; SIT: 9.8 mm; meibum grade was 1. The fifth row shows the left eye of a 63-year-old male in the DM + DE group with moderate glycemic control: HbA_1*c*_: 7.2%; OSDI: 25; BUT: 4.4 s; SIT: 7.2 mm; meibum grade was 1. The sixth row shows the right eye of a 64-year-old female from the DM + DE group with poor glycemic control: HbA_1*c*_: 9.5%; OSDI: 34; BUT: 3.1 s; SIT: 5.2 mm; meibum grade was 3. White arrows: lid margin engorgement; red arrows: abnormal secretion of meibum.

The parameters of tear film and meibomian gland in the two DM − DE subgroups were also different at different HbA_1*c*_ levels, but the SIT value were still within the normal range. In the subgroup with good glycemic control (DM − DE-1), the BUT and LLT scores were significantly lower, and meibomian gland parameters were significantly higher when compared with the NC group (*p* < 0.001). In the subgroup with poor glycemic control (DM − DE-2), the BUT, LLT, and FL scores were significantly altered when compared to those of the NC group (*p* < 0.001). All meibomian gland parameters of these two subgroups were dramatically higher when compared to those of the NC group (*p* < 0.001). These results suggested that patients with diabetes might had a state of asymptomatic MGD even though the blood glucose levels were well controlled (HbA_1*c*_ < 7%). Asymptomatic MGD might already exist in T2D with good glycemic control but without dry eye. Representative images from patients in each subgroup are shown in Figure 2.

### Correlations Between HbA_1*c*_ Levels and Parameters of Tear Film and Meibomian Gland in Type 2 Diabetics

In the DM group, the Spearman correlation analysis showed that the HbA_1*c*_ level was positively correlated with the OSDI score (*R* = 0.644, *p* < 0.001) and FL score (*R* = 0.393, *p* < 0.001) but was inversely correlated with SIT (*R* = − 0.586, *p* < 0.001), tear BUT (*R* = − 0.575, *p* < 0.001), and LLT (*R* = − 0.560, *p* < 0.001) values. The analysis showed that the HbA_1*c*_ level was positively correlated with all the four meibomian gland parameters (lid margin score, *R* = 0.427; MGYLS score, *R* = 0.407; meibum score, *R* = 0.452; and meiboscore, *R* = 0.454; all *p* < 0.001). Among the relevant variables (i.e., age, blood glucose, HbA_1*c*_, and duration of diabetes), the multiple linear regression analysis revealed that only HbA_1*c*_ level was significantly associated with meibum score (β = 0.397, *p* = 0.036) in the DM group, whereas age was significantly associated with meiboscore (β = 0.362, *p* = 0.001) and MGYLS score (β = 0.310, *p* = 0.003). Blood glucose level was not associated with any of the meibomian gland parameters (*p* > 0.05).

### Meibomian Gland Dysfunction in Type 2 Diabetics With Long or Short Duration

Patients in the DM group were further separated into three subgroups based on the duration of the diabetes. The parameters of tear film and meibomian gland in these subgroups are shown in Table 5. The OSDI score, FL score, the lid margin score, MGYLS score, the meibum score, and meiboscore in the long and moderate duration groups were significantly higher, whereas the SIT value was significantly lower (*p* < 0.001) than that of the short duration group (*p* < 0.01). No significant difference was found in these parameters between the moderate duration group and long duration group. Interestingly, as the duration of diabetes increased from moderate to long duration, the value of BUT and LLT also decreased (*p* < 0.001), but the four MGD parameters did not deteriorate accordingly (*p* > 0.05). Multiple linear regression analysis also showed that the diabetic duration was significantly associated with meiboscore (β = 0.334, *p* = 0.002), the lid margin score (β = 0.498, *p* = 0.000), and the MGYLS score (β = 0.355, *p* = 0.001) in the DM group.

**TABLE 5:** Tear film and meibomian gland parameters in long or short durations of diabetes.

	Short duration (≤5 years, *n* = 52)	Moderate duration (>5, ≤10 years, *n* = 32)	Long duration (>10 years, *n* = 15)	*p* value
Course (years)	3.48 ± 1.36	7.55 ± 1.32^a^	12.75 ± 1.83^ab^	<0.001
OSDI	22.62 ± 4.71	27.94 ± 4.51^a^	34.88 ± 4.67^a^	<0.001
Schirmer I (mm)	10.45 ± 2.53	6.85 ± 1.41^a^	4.81 ± 1.52^a^	<0.001
BUT(s)	6.68 ± 1.70	5.23 ± 1.58^a^	3.45 ± 1.00^ab^	<0.001
FLscore	0.48 ± 0.59	1.08 ± 0.57^a^	1.38 ± 0.50^a^	<0.001
LLT (nm)	61.16 ± 6.24	53.70 ± 5.66^a^	46.81 ± 5.58^ab^	<0.001
Lid margin score	1.87 ± 0.53	2.55 ± 0.60^a^	2.94 ± 0.77^a^	<0.001
MGYLS score	5.77 ± 1.46	7.58 ± 1.55^a^	8.81 ± 1.68^a^	<0.001
Meibum score	23.61 ± 4.98	29.43 ± 4.52^a^	32.81 ± 4.32^a^	<0.001
Meiboscore	3.21 ± 0.85	4.25 ± 0.78^a^	4.81 ± 0.66^a^	<0.001

*n, number of participants; OSDI, ocular surface disease index; BUT, tear breakup time; FL, corneal fluorescein staining; LLT, lipid layer thickness; MGYLS, meibomian gland yielding liquid secretion. All data are presented as mean ± standard deviation. ^a^p < 0.05 compared to the short duration group. ^b^p < 0.05 compared to the moderate duration group.*

## Discussion

This study demonstrated that patients with T2D have apparent ocular discomfort and dry eye with slightly reduced aqueous tear volume. Meibomian gland and tear function were impaired in patients with T2D and deteriorated with moderate or long diabetic duration and a high HbA_1*c*_ level. The values of four meibomian gland parameters and the FL score were significantly lower in patients with T2D without dry eye than those with T2D with dry eye. More importantly, the values of four meibomian gland parameters were found to be significantly higher in patients with T2D without dry eye than the healthy population, although the OSDI scores were all below 13. Our study indicated that asymptomatic MGD may emerge before the ocular discomfort and dry eye develops in patients with T2D.

Previous studies suggested that tear deficiency might be the main cause of dry eye in T2D (25–27), whereas some researchers found no significant difference in tear volume between patient with T2D and the normal population (28, 29). Our results indicated that, even though the SIT value of the DM group was somewhat lower than that of the NC group, the tear production was still close to the cut-off value (10 mm). In patients with T2D, the subgroup with good glycemic control (HbA_1*c*_ < 7%) had normal SIT value, whereas the subgroup with poor glycemic control (HbA_1*c*_ ≥ 9%) showed reduced SIT value. The HbA_1*c*_ levels were inversely correlated with tear secretion volumes. In most of the previous studies, tear volume data were analyzed without subdivision on diabetic condition, thus probably leading to different conclusions.

Previous studies revealed a high incidence of MGD in patients with T2D (30–32). However, the role of MGD in the pathogenesis of dry eye in patients with T2D remains unclear. Our results showed the values of four meibomian gland parameters were significantly higher in the DM − DE group than the NC group, although the OSDI scores were below 13 in both groups. This suggests that asymptomatic MGD might emerge before the ocular discomfort develops. Some differences in clinical features were also noted in the meibomian gland and tear film parameters between the DM − DE and DE groups and between the DM + DE and DE groups. The values of MGD parameters were significantly higher (*p* < 0.05), whereas the LLT score was lower (*p* < 0.05) in the DM + DE group than that of the DE group. On the other hand, the MGYLS score in the DM − DE group was higher even in subjects who presented no obvious ocular discomfort when compared with the DE group. The dry eye condition in T2D might mainly originate from the impairment of meibomian gland function (Table 3). Multivariate analysis also showed that the HbA_1*c*_ level was significantly associated with the meibum score, whereas the duration of diabetes was associated with meiboscore, lid margin score, and MGYLS score. Taken together, asymptomatic MGD may be an early sign of dry eye in T2D.

The mechanism of MGD in patients with diabetes is still unclear. Clinical studies revealed that peripheral neuropathy and corneal hypoesthesia are associated with declines in nerve impulses emanating from the brain and lead to reduced blink rates (27, 33, 34). Therefore, it is speculated that corneal hypoesthesia leads to the decline in blinking rate, and then, the driving forces that result in the eventual delivery of meibum are weaker (6). Reduced meibum destabilizes the tear film and finally aggravates tear evaporation in patients with T2DM. However, further investigation is needed for the detailed mechanisms of MGD in patients with T2DM.

The HbA_1*c*_ has been recognized as the “golden standard” for monitoring glycemic control (35). Our study described the association between HbA_1*c*_ level and meibum score. A previous study also found that the HbA_1*c*_ levels correlated with dry eye in patients with diabetes (4, 9). Dogru et al. (25) found that the ocular symptoms of diabetes are related with poor metabolic control and peripheral neuropathy. Prolonged hyperglycemia could inhibit conjunctival goblet cell proliferation and corneal nerve conduction and cause decline of tear volume and stability. Seifart and Strempel (36) also found that the HbA_1*c*_ level was associated with the incidence of dry eye. However, blood glucose level was not a significant influence factor of the meibomian gland parameters in our study. The duration of diabetes was more likely to affect meiboscore, lid margin score, and MGYLS score, whereas the HbA_1*c*_ level was more likely to affect the meibum score. In our data, as the duration of diabetes increased from moderate to long duration (Table 5), the values of BUT and LLT also decreased (*p* < 0.001), but the four MGD parameters did not deteriorate accordingly (*p* > 0.05). This implied that there would be other aggravating factors of tear film stability besides MGD for patients with DM in the long duration group. Further studies are needed to investigate the molecular role of HbA_1*c*_ in the development of MGD.

Our data showed the impaired meibomian gland function in patients with T2D without ocular discomfort. In fact, a lack of association between symptoms and signs has also been reported in other ocular surface disorders in several studies (37). Our data suggests the need to perform meibomian gland evaluation to detect the presence of asymptomatic or symptomatic MGD in patients with T2D even with a good HbA_1*c*_ level and a short diabetic duration. The measurement of HbA_1*c*_ appears to be more meaningful than the blood glucose level in the evaluation and management of dry eye and MGD in T2D. On the other hand, the aggravation of MGD in T2D may imply to some extent the poor glycemic control. In the future study, it would be important to identify whether preclinical features are likely to be predictive of progressive diseases and whether early treatment might delay progression or reverse these pathologic events. Treatment for early-stage disease is relatively simple. Our data provided good reasons to offer treatment at an early, preclinical stage of the disease, such as type 2 diabetes and dry eye.

Limitations of our study include a relatively small sample size that may reduce the power to further interpretation. Meanwhile, the lack of information, such as the usage of anti-diabetic agents, the blood lipid level, body weight and diet, visual terminal use, sleep time, and emotional status, may hinder generalizability of this cross-sectional study. The lack of application of some techniques for meibomian gland evaluation, such as optical coherence tomography and *in vivo* confocal microscopy (23, 38), may also leave us out of some important findings. In addition, this study includes Chinese population only, which may also limit the ability to apply our results to other races. Furthermore, patients with blepharitis, history of ophthalmic surgery, and continuous ophthalmic topical medications were excluded from this study, as they always suffer from severe MGD and dry eye. In fact, the role of MGD in the pathogenesis of dry eye may be underestimated by the exclusion of these patients with or without type 2 diabetes. Another major limitation is that our findings cannot prove causation and successively relation as these data are observational.

## Conclusion

In summary, patients with T2D suffer from ocular surface discomfort and dry eye with slight aqueous tear volume reduction, whereas the morphology and function of meibomian glands have significantly changed. More importantly, asymptomatic MGD has emerged in patients with T2D even with good glycemic control. The tear function and meibomian gland parameters deteriorated in patients as the diabetic duration became longer and the glycemic control became worser. Asymptomatic MGD may be an early sign of dry eye and ocular discomfort in patients with T2D.

## Data Availability Statement

The original contributions presented in the study are included in the article/supplementary material, further inquiries can be directed to the corresponding authors.

## Ethics Statement

The studies involving human participants were reviewed and approved by the Ethics Committee of Xiamen Eye Center. The patients/participants provided their written informed consent to participate in this study.

## Author Contributions

ZLn and ZLu: conceptualization and writing—review and editing. HW, XF, and SL: methodology. XS, ZLn, and ZLu: validation. HW and XF: formal analysis. XX, ZX, ND, and SL: investigation. SL and XF: data curation and original draft preparation. All authors have read and agreed to the published version of the manuscript.

## Conflict of Interest

The authors declare that the research was conducted in the absence of any commercial or financial relationships that could be construed as a potential conflict of interest.

## Publisher’s Note

All claims expressed in this article are solely those of the authors and do not necessarily represent those of their affiliated organizations, or those of the publisher, the editors and the reviewers. Any product that may be evaluated in this article, or claim that may be made by its manufacturer, is not guaranteed or endorsed by the publisher.
